# What Drives Task Performance During Animal Fluency in People With Alzheimer’s Disease?

**DOI:** 10.3389/fpsyg.2020.01485

**Published:** 2020-07-21

**Authors:** Adrià Rofes, Vânia de Aguiar, Roel Jonkers, Se Jin Oh, Gayle DeDe, Jee Eun Sung

**Affiliations:** ^1^Center for Language and Cognition Groningen (CLCG), University of Groningen, Groningen, Netherlands; ^2^Department of Communication Disorders, EWHA Womans University, Seoul, South Korea; ^3^Department of Communication Sciences and Disorders, Temple University, Philadelphia, PA, United States

**Keywords:** fluency, category, animal, switches, clusters, age of acquisition

## Abstract

**Background:**

Animal fluency is a widely used task to assess people with Alzheimer’s disease (AD) and other neurological disorders. The mechanisms that drive performance in this task are argued to rely on language and executive functions. However, there is little information regarding what specific aspects of these cognitive processes drive performance on this task.

**Objective:**

To understand which aspects of language (i.e., semantics, phonological output lexicon, phonological assembly) and executive function (i.e., mental set shifting; information updating and monitoring; inhibition of possible responses) are involved in the performance of animal fluency in people with AD.

**Methods:**

Animal fluency data from 58 people with probable AD from the DementiaBank Pittsburgh Corpus were analyzed. Number of clusters and switches were measured and nine word properties (e.g., frequency, familiarity) for each of the correct words (i.e., each word counting toward the total score, disregarding non-animals and repetitions) were determined. Random forests were used to understand which variables predicted the total number of correct words, and conditional inference trees were used to search for interactions between the variables. Finally, Wilcoxon tests were implemented to cross-validate the results, by comparing the performance of participants with scores below the norm in animal fluency against participants with scores within the norm based on a large normative sample.

**Results:**

Switches and age of acquisition emerged as the most important variables to predict total number of correct words in animal fluency in people with AD. Cross-validating the results, people with AD whose animal fluency scores fell below the norm produced fewer switches and words with lower age of acquisition than people with AD with scores in the normal range.

**Conclusion:**

The results indicate that people with AD rely on executive functioning (information updating and monitoring) and language (phonological output lexicon, not necessarily semantics) to produce words on animal fluency.

## Introduction

Fluency tasks are commonly used to assess people with different neurological disorders, including people with Alzheimer’s disease (AD) (e.g., Monsch et al., 1992; Cohen et al., 1999; Quinn et al., 2012; Shao et al., 2014; Rodrigues et al., 2015; Rofes et al., 2017, 2019). Typically, researchers and clinicians ask participants to name as many words as possible starting with a common category (e.g., animals, fruits, vegetables) or with a common letter (e.g., F, A, S). In this paper, we focused on the strategies that people with AD use to respond to category fluency and, specifically, to animal fluency.

Animal fluency consists of naming as many animals as possible in a short period of time (typically, 1 min). This task is included in many clinical screenings and has been used for a long time (e.g., Henry and Crawford, 2004; Henry et al., 2004; Laws et al., 2010). Also, it has been shown to discriminate people with mild cognitive impairment (MCI) and dementia from people with normal aging (e.g., Choi, 2008; cf. Moreno-Martínez et al., 2017; Oh et al., 2019). In people with AD, animal fluency is often more impaired than letter fluency (e.g., Monsch et al., 1992; Henry and Crawford, 2004; Henry et al., 2004), though the opposite pattern has also been reported (e.g., Fisher et al., 2004; Laws et al., 2010).

Scores below the norm in animal fluency are thought to reflect damage to language and executive functions (e.g., Sauzéon et al., 2011; Shao et al., 2014; Takács et al., 2014). This is because the task requires word retrieval (i.e., to say as many animals as possible) and because participants need to meet certain constraints when retrieving the words (e.g., using only animals, not repeating words, avoiding proper nouns). Indeed, people with good language capacities, including large vocabularies, and normal executive functioning tend to produce more names of animals than those with smaller vocabularies (e.g., Sauzéon et al., 2011). Also, people with problems in executive functions, and not necessarily in language, such as children with attention deficit hyperactivity disorder, produce fewer words in animal fluency than matched-controlled pairs (e.g., Takács et al., 2014).

Nonetheless, the mechanisms that drive task performance in animal fluency are under debate. It is unclear which aspects of language (i.e., semantic level, phonological output lexicon, phonological assembly) and executive function (i.e., mental set shifting, information updating and monitoring, inhibition of responses) may be involved in the performance of animal fluency, and to what extent. To address this question, this paper investigates the number of switches and clusters in animal fluency, as well as a relatively wide number of word properties, such as frequency, age of acquisition, concreteness. The novelty of our work lays in two factors: (1) focusing on a large number of variables extracted from the task itself, as opposed to studying external tasks; and (2) exploring multiple variables at the same time using two machine learning algorithms, random forests and conditional inference trees (e.g., Breiman, 2001; Strobl et al., 2008).

In the following sections, we outline three different approaches to studying the drivers (or determinants) of performance on fluency tasks. These are (1) correlations of fluency tasks with other tests of language and executive functions, and the study of variables that can be extracted from fluency tasks, namely, (2) switches and clusters, and (3) word properties. Finally, we describe the aims and predictions of this study.

### Correlations of Fluency Scores With Other Tests

Henry et al. (2004) meta-analysis suggested that animal fluency is impaired in people with AD due to a degradation of semantic associations within the lexicon. Further, the authors stressed the role of executive functions (i.e., monitoring and inhibition) in verbal fluency tasks. Their rationale was that animal fluency scores were significantly lower than object naming scores, which require less effortful retrieval. In another study, Whiteside et al. (2016) used factor analysis to examine relationships among several tests of language, including verbal fluency, and executive functions in people with traumatic brain injury, multiple sclerosis, or dementia. The results showed that animal fluency is primarily influenced by language rather than executive functions, but the authors did not exclude possible influences of executive functions.

Shao et al. (2014) looked at the fluency scores of healthy older individuals and also at other tests tapping language and executive functions. In contrast to Henry et al. (2004) and Whiteside et al. (2016), they found that animal fluency performance was influenced by updating ability (i.e., solving mathematical problems and memorizing lists of words), vocabulary size (i.e., matching a word with a set of possible descriptions), and speed of lexical access (i.e., mean reaction times in picture naming). Consequently, the authors argued that animal fluency has a “hybrid” profile, meaning that it taps into both language and executive functions. Consistent with the idea of “hybrid” profile, neuroimaging studies have attributed good performance on verbal fluency to (posterior) temporal areas that are key for lexical–semantic processing (e.g., Gourovitch et al., 2000; Henry and Crawford, 2004), and to frontal and inferior parietal cortex, which are involved in executive functions (e.g., Vonk et al., 2018).

### Variables Extracted From Fluency Tasks: Clusters and Switches

The use of variables, such as clusters, switches, and word properties in fluency tasks most likely emerged because total number of words is too coarse a measure to reveal why participants perform within or below the norm, and also because low scores may be driven by different impairments in language and executive functions. The motivation of this approach is capturing both how well a participant performs and how that performance is achieved (e.g., Kaplan, 1990).

Task performance in animal fluency involves the retrieval of words grouped into subcategories (e.g., Raskin et al., 1992; Troyer et al., 1997; Troyer, 2000; Abwender et al., 2001). Common subcategories include human use (e.g., farm animals), living environment (e.g., African animals), and zoological taxonomy (e.g., feline, bovine). Hence, participants search for subcategories of animals and then generate as many words as possible within the subcategory. Clusters refer to successively generated words that belong to the same semantic family and that can be subcategorized under an umbrella category. For example, according to Troyer et al. (1997)’s criteria, there are two clusters in a sequence like “cow, sheep, horse, donkey, lion, tiger.” One cluster corresponds to farm animals (from “cow” to “donkey”) and another cluster corresponds to felines (from “lion” to “tiger”). Cluster size is calculated based on the number of words with a subcategory minus one, as clusters of only one item are not counted. Thus, the cluster sizes for farm animals and felines in the example above are 3 and 1, respectively. Cluster size has been related to semantic memory impairment, as people with lesions in the temporal lobe produce smaller clusters than healthy older individuals (Troyer et al., 1998b). Also, people with AD produce smaller cluster sizes than people with Parkinson’s disease (with and without dementia) and healthy older individuals (Troyer et al., 1998a).

Switches represent changing from one subcategory to another (e.g., Troyer et al., 1997; Abwender et al., 2001). Switching occurs when participants exhaust their ability to generate words within a subcategory, even if the subcategory only contains one word. Thus, the participant stops naming items in that subcategory and moves to another subcategory. The example above (i.e., “cow, sheep, horse, donkey, lion, tiger”) contains one switch, when the subcategory farm animals is replaced by the subcategory felines.

Number of switches has been argued to relate to different aspects of executive functioning. Following the theoretical framework of Miyake et al. (2000) we will argue that switching reflects two aspects of executive functions: information updating and monitoring. This is because changing from one subcategory to another requires active renewal of the criteria used to search words (vs. passively storing words, as in a word learning task). Also, switching between subcategories requires keeping track of the responses that were already given, while adhering to the task instructions (e.g., all words need to be animals, proper nouns are not allowed). We do not consider switches to reflect inhibition because they do not reflect controlled suppression of responses. In contrast, a typical measure of inhibition is the picture word interference paradigm, in which participants are asked to name an object with a semantically related word written on top (e.g., Shao et al., 2015).

Other authors discussed inhibition in the context of fluency tasks (e.g., Henry et al., 2004; Takács et al., 2014). This is because naming words in a specific category requires disregarding other words that may be activated but that do not meet criteria (Ellis and Lambon Ralph, 2000). However, this type of inhibition is an automatic process that is active throughout the task, rather than the controlled suppression of responses that is necessary for a switch to occur. We also do not consider switches to relate to mental set shifting because this executive function reflects the ability to shift back and forth between different tasks. That is, mental set shifting involves active disengagement of one task to engage in another task (e.g., Miyake et al., 2000). In contrast, switches occur due to exhaustion in the generation of more items within one subcategory and, arguably, within the same task.

It is worth noting that clusters and switches do not provide pure measures of executive function or lexical retrieval. Mayr (2002) indicated that the number of switches may reflect both executive functioning and lexical retrieval. On their account, number of switches partially reflects the time it takes to update the criterion to generate a new subcategory. This function is arguably dependent on executive functions, as it requires information updating and monitoring. Mayr (2002) also suggested that number of switches partially reflects the participant’s ability to retrieve exemplars within a subcategory. This function is arguably more dependent on lexical/semantic abilities, as it is requires searching for words in the lexicon.

Additionally, there are expansions of the analysis of clusters and switches. For example, Johns et al. (2018) used a computational cognitive model trained on a large linguistic corpus. The model used multiple sources of information and indicated that word frequency and order (i.e., role of words with respect to other words, e.g., *cat* and *panther* pounce on prey) increase over time in people before being diagnosed with MCI (pre-MCI) compared to people without MCI. In a similar study, Taler et al. (2019) found that healthy older adults produced words of denser semantic neighborhood and higher frequency than healthy younger adults. Quaranta et al. (2019) stressed the relevance of semantic pairwise similarity, particularly in identifying people with MCI who convert to dementia from healthy individuals. In the present study, a measure of semantic association called Latent Semantic Analysis (LSA) was used (Günther et al., 2015). The added value of computational methods over the use of clusters and switches lays, among other things, on the fact that computational methods are less dependent on subjective ratings; hence, the results are easier to replicate, faster to obtain, and less prone to human error.

### Variables Extracted From Fluency Tasks: Word Properties

In addition to clusters and switches, word properties or psycholinguistic variables can reveal which aspects of language are most relevant during performance of verbal fluency tasks and the underlying language impairments that lead to poor task performance (cf. Cutler, 1981; Whitworth et al., 2014; Rofes et al., 2019; Alyahya et al., 2020).

Difficulties associated with familiarity, imageability, concreteness, and semantic association are indicative of impairments of the semantic level, that is, the store of meanings that are activated in response to an idea or concept (e.g., Nickels and Howard, 1994, 1995; Rofes et al., 2019). Semantic level impairments can affect production and comprehension of spoken and written words (Whitworth et al., 2014). Familiarity is measured by asking people how often they are in contact with or use certain words (e.g., “vertex” would be low in familiarity, while “zebra” would be high; Noble, 1953). Imageability is obtained by asking people the degree to which a word evokes a sensory experience or mental image. For example, “hope” is low in imageability, while “house” is high; Paivio et al., 1968). Concreteness indicates the degree to which a concept refers to a perceptible entity (e.g., “couch” is a concrete word, while “ideal” is an abstract word; Paivio et al., 1968). Finally, semantic association is obtained with corpora and indicates the strength of the relationship between two words in a corpus. For example, “monkey” and “banana” are closer to one another and thus more semantically associated than “monkey” and “pasta” (Günther et al., 2015).

Word frequency, age of acquisition, and phonological/orthographic similarity are associated with the phonological output lexicon, which is a store of spoken word forms (e.g., Gilhooly and Watson, 1981; cf. Nickels and Howard, 1995; Brysbaert et al., 2000; Cuetos et al., 2010). Impairments associated with these word properties may be due to difficulty accessing the phonological output lexicon from semantics or impairments in the phonological output lexicon itself. Damage to the phonological output lexicon affects oral naming, and speaking, but not written production, or spoken or written comprehension. Frequency ratings are obtained from large corpora and indicate how many times a word appears in a corpus (e.g., “child” is more frequently occurring than “tyke”; Baayen et al., 1995). Age of acquisition is typically obtained using questionnaires where people are asked when they learned a word in the spoken or written form. For example, “ball” and “door” typically are learned early in life, whereas “metropolitan” and “manuscript” are learned later in life (Stadthagen-Gonzalez and Davis, 2006; Juhasz et al., 2015). Phonological and orthographic similarity are two independent measures of lexical neighborhood that are obtained by counting the number of words that can be formed by substituting one phoneme/letter of the target word in a given corpus. For example, “soul” has many phonologically similar neighbors, including “bowl, “coal”, and “dole.” In contrast, “mountain” has only one phonologically similar neighbor, “fountain” (Davis, 2005).

Finally, length in phonemes is a word property that can reflect issues in phonological encoding/assembly. Phonological encoding corresponds to the concatenation of phoneme strings in preparation for conversion into neuromuscular commands for articulation (e.g., Caramazza et al., 1986; Shallice et al., 2000; Nickels and Howard, 2004). Length is obtained by counting the number of phonemes in a word (e.g., “cat” has 3 phonemes, while “uncopyrightable” has 13 phonemes).

The literature regarding word properties and fluency tasks is growing. Some groups have looked into characterizing and classifying individuals with AD and other types of dementia from people without brain damage. For example, we recently found that familiarity was particularly relevant to classifying individuals with semantic variant primary progressive aphasia (svPPA), relative to people with logopenic variant PPA (lvPPA), non-fluent variant PPA (nfvPPA), and people without brain damage (Rofes et al., 2019). In Marczinski and Kertesz (2006), it is reported that people with svPPA and AD produce fewer and more frequent words than healthy people in word fluency tasks. However, another study found that people with svPPA and AD do not differ in terms of the types of words they produce in verbal fluency tasks (Vonk et al., 2019b).

Other groups have looked at word properties as a way to predict the occurrence of AD. In one study, word frequency was relevant to identify individuals with genetic markers that increase their likelihood of developing AD (i.e., apolipoprotein E). These individuals produced words of higher frequency, as opposed to individuals without those genetic markers (Vonk et al., 2019a). Clark et al. (2014) indicated that semantic similarity (word co-occurrence given a large written corpus, e.g., “dog” and “cat” can be found closer together in a corpus, than “dog” and “quinoa”) was relevant to identify individuals with AD, particularly in animal fluency. Vita et al. (2014) found that people with MCI and people with AD produced words with higher typicality compared to healthy individuals, and that typicality (e.g., “pigeons” are more representatives of the category “bird” than “ostriches”) was the best predictor of people with MCI progressing to AD. Other researchers showed that age of acquisition is the best predictor of disease severity in people with AD. Also, that in comparison to people without brain damage, people with AD produce items that are acquired earlier in life, as well as items that are more frequent, more typical, and shorter in length (Forbes-McKay et al., 2005). A similar pattern was reported for people with MCI, as these individuals produce early acquired and more familiar words than healthy individuals (Biundo et al., 2011). In this same study, a particular emphasis was paid to age of acquisition, as people with MCI that were e4 carriers and later on developed AD produced more words that were earlier acquired than people with MCI who were not e4 carriers (Biundo et al., 2011). Other studies have stressed the role of age of acquisition in tasks other than verbal fluency (Cuetos et al., 2010).

### Aims and Predictions

The current study is a data-driven attempt to understand what linguistic and executive factors influence performance (as measured with the total number of words) on animal fluency in people with AD. Based on previous reports, we expect linguistic variables that relate to the semantic level and the phonological output lexicon (i.e., clusters, frequency, imageability, concreteness, familiarity, age of acquisition, semantic association, orthographic similarity, and phonological similarity) to be more predictive of total number of words than number of switches, which reflect executive functions related to information updating and monitoring (i.e., switches). Given the differences in the inclusion of these variables as well as different results in previous studies, it is hard to establish which (if any) of these linguistic variables may be superior at driving task performance in animal fluency.

## Materials and Methods

### Participants

The data of 58 native English speakers with probable AD was extracted from DementiaBank Pittsburgh corpus (Becker et al., 1994). The DementiaBank is a shared database for the study of communication in dementia. It is supported by NIH-NIDCD grant R01-DC008524. The participants were 42 women and 16 men, with a mean age of 72 years (*SD* = 8.8, range = 56–88) and a mean education of 12 years (*SD* = 3, range = 6–20). Participants’ scores on the Mini Mental Stage Examination (Folstein et al., 1975) averaged 19.07 (*SD* = 4.04, range = 10–27). Table 1 presents demographic data.

**TABLE 1:** Demographics, total words, and mean values for clusters, switches, and word properties for animal fluency in our sample of people with AD.

	**Age**	**Age.O**	**Edu**	**MMSE**	**Words**	**CL_CR**	**SW_CR**	**Freq**	**Imag**	**Fam**	**Conc**	**AoA**	**LSA**	**LP**	**OS**	**PS**
Mean	72.16	68.09	11.71	19.07	8.79	0.60	6.96	33.07	618.44	532.36	4.91	4.38	0.57	4.00	10.20	13.72
*SD*	8.80	8.53	2.70	4.04	4.56	0.83	3.71	13.41	7.44	23.72	0.04	0.60	0.14	0.81	3.59	4.52

*Std, Standard deviation; Age, Age at assessment time; Age.O, Age of AD diagnosis; Edu, Education in years; MMSE, Minimental stage examination scores; Words, Total number of words; CL_CR, Corrected clusters; SW_CR, Corrected Switches; Freq, Word frequency; Imag, Word Imageability; Fam, Word Familiarity; Conc, Word concreteness; AoA, Word Age of Acquisition; LSA, Semantic association (i.e., Latent Semantic Analysis); LP, Length in phonemes; OS, Orthographic similarity; PS, Phonological similarity.*

### Tasks, Scoring, and Reliability

Participants were given 60 seconds to produce as many words as possible belonging to the category “animals.” Similar to other studies, participants were told not to produce proper nouns. The criteria outlined in Troyer (2000) were used to score the tasks for number of switches and mean cluster size. Clusters were identified as successively generated words belonging to the same semantic subcategories (i.e., African animals, Australian animals, Arctic/Far North animals, farm animals, North American animals, water animals, beasts of burden, animals used for their fur, pets, birds, bovine, canine, deers, feline, fish, insects, insectivores, primates, rabbits, reptiles/amphibians, rodents, weasels). In the case where two categories overlapped, with some items belonging to both categories, the overlapping items were assigned to both categories. In the case where smaller clusters were embedded within larger ones, only the larger common category was used. Cluster size is defined as the number of items within a subcategory minus one, because clusters must contain more than one member. The mean cluster size was computed by summing the size of each cluster and dividing it by the number of clusters. The number of switches was calculated by counting the number of transitions from one cluster to another.

Nine word properties were extracted for each correct word, that is, each word counting toward the total score disregarding non-animals and repetitions. The word properties we studied were frequency, imageability, concreteness, familiarity, age of acquisition, semantic association, length in phonemes, orthographic similarity, and phonological similarity. References for each of the databases we used can be found in Supplementary Material.

The scoring of switches/clusters was performed by two doctoral students and two master’s students majoring in speech-language pathology. Before the analysis, all the raters reviewed the scoring system of Troyer (2000) and practiced rating until inter-rater reliability became 100%. The extraction of word properties was performed by one of the authors (AR) using the computer program N-Watch (Davis, 2005) with updated databases for imageability, familiarity, concreteness, and age of acquisition.

### Analyses

First, to understand how many individuals with AD had scores below the norm in animal fluency, we compared the scores of each individual to the appropriate normative sample using data from Tombaugh et al. (1999). Tombaugh et al. (1999) data include 735 participants, including nine subgroups defined by age and education, to allow matched comparisons with each of the people with AD. The subgroups include participants from 16 to 95 years of age (divided into three groups: 16–59, 60–79, and 80–95) and education ranges from 0 to 21 (divided into three groups: 0–8, 9–12, and 13–21). For the purposes of this study, we used all subgroups with the exception of age 16–59 and education 0–8 years. The number of participants in the subgroups ranged from 46 to 292 (mean = 160; *SD* = 98). This approach was deemed better than collecting new data, given the large number of participants in the database. Data for each individual with AD were compared to the appropriate normative sample with modified *t*-tests, using the computer program Singlims_ES (Crawford et al., 2010). Modified *t*-tests allow us to assess whether the scores of each participant significantly differ from a control or normative sample.

Second, to understand the influence of cluster size, number of switches, and nine word properties on the responses of people with AD, we used a machine learning algorithm, namely, random forests. Random forests are suitable when sample sizes are small and there are many predictor variables, some of which may be collinear (Breiman, 2001; Strobl et al., 2008). The sample size in the present study is relatively small (*n* = 58). Further, we entered 11 variables in the prediction models, some of which are known to correlate with one another (e.g., frequency with age of acquisition; concreteness with imageability). We used random forests for regression and “variable selection”, that is, to choose which variables function as better predictors of the total number of words in animal fluency in our sample. Thus, random forests identify which variables best account for the total number of words in a fluency task in people with AD.

Random forest analyses were completed using the program R. We performed the following three analysis steps: (1) We generated a random forest with unbiased conditional inference trees (Strobl et al., 2008), using the cforest function (Hothorn et al., 2017). (2) Using the varimp function (Hothorn et al., 2017), we extracted the relative importance of each predictor using conditional permutation variable importance (Strobl et al., 2008). Importance reflects how well each variable predicts the dependent variable (i.e., total number of words in animal fluency). When removing a given variable from the model results in a decrease in model prediction accuracy, that variable is ranked highly in terms of importance (Strobl et al., 2008). (3) Finally, we estimated predictor accuracy including only potentially informative predictors using leave-one-out cross-validation. In this procedure, the classifier is trained on a dataset in which one data point (i.e., one participant) is omitted. The value of the omitted observation is then predicted and saved. This procedure is repeated for each data point. Then, we examined the relation between the actual values and the predicted values of total number of words in animal fluency. This way, we evaluated the accuracy of predictions, measured in terms of *R*^2^, root mean squared error (RMSE), and mean absolute error (MAE). For further information on this methodology, see Tagliamonte and Baayen (2012) and Zhang and Min (2016). Sample R scripts can be found in de Aguiar et al. (2015).

Random forest analyses are used to select variables, but they do not examine interactions among variables. Thus, we used another machine learning algorithm, conditional inference trees, to understand how variables interact. The conditional inference tree algorithm performs statistical tests to identify points along the scale of a variable where the prediction values of the dependent measure change significantly (i.e., split points). The end result of this algorithm is a tree-like representation, with nodes representing split points for variables that are significant. Conditional inference trees, for example, could indicate that mean age of acquisition scores above 6 (on a scale of 1–10) and a greater number of switches may be predictive of high number of words in animal fluency.

Finally, to cross-validate the results using a different statistical approach, we compared the total number of words in animal fluency for individuals with AD who performed below and within the normal range (based on modified *t*-tests) on animal fluency. To do so, we ran Wilcoxon tests with the independent variable group (below normal vs. within normal) and the dependent variable to those variables shown to be relevant in the conditional inference trees. We used non-parametric statistics because the data were not normally distributed, as indicated by the Shapiro test. In Supplementary Material, we provided the data we used in this study.

## Results

In Table 1, an overview of our sample is provided, including demographic information, total number of words and mean values for cluster size, number of switches, and word properties. In the appendices, a detailed table with the same information for each participant is provided. Overall, 35 of 58 people with AD (60.3%) produced significantly fewer words in animal fluency compared to the normative sample of Tombaugh et al. (1999).

The random forests regression model, which was computed to select variables ranking high in importance, explained 60% of variance in the dependent measure *total number of words* (*R*^2^ = 0.6; RMSE = 2.87; MAE = 2.15) after leave-one-out cross-validation. Also, the most informative variables in the regression of total number of words were, in order of higher to lower importance: number of switches, age of acquisition, frequency, familiarity, orthographic similarity, phonological similarity, length in phonemes, and mean cluster size.

**FIGURE 1:**
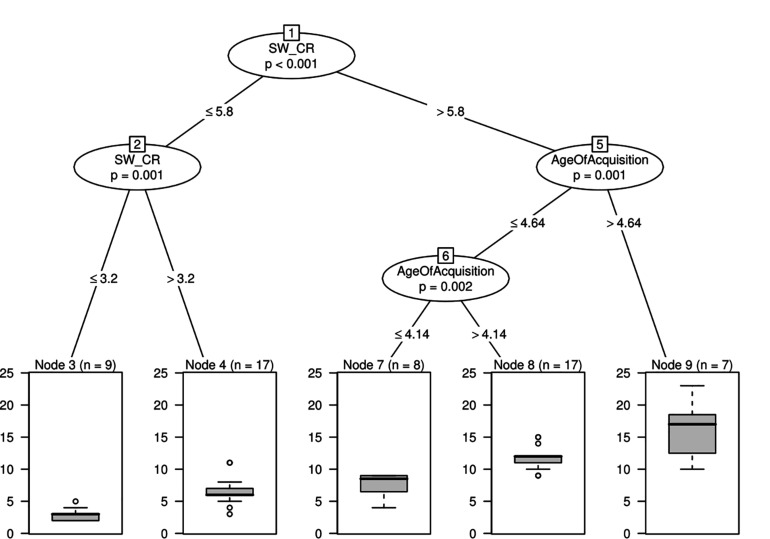
Conditional inference tree with switches and age of acquisition as relevant variables. Conditional inference tree representing how variables interact. Each circle or box represents a node. The number of each node is written on top of the circle or box. Nodes represented with circles show variables that are significantly associated with different distributions of the value of the dependent variable, *total number of words*, and include a *p*-value. Below each circle, the values indicate a point in each variable where individuals split into those groups. The nodes represented by boxes are the terminal nodes, which describe a subsection of the sample that corresponds to that split in the data and provides a plot representing the distribution of the data for each subsection of the sample. SW_CR, Switches Correct.

Conditional inference trees computed by using the variables shown as important in random forests identified an interaction between switches and age of acquisition (Figure 1) (*R*^2^ = 0.46; RMSE = 3.33; MAE = 2.45). The split point at the highest node of the tree (node 1, for the variable corrected number of switches) indicates that participants with more than 5.8 switches (*n* = 32, combining the nodes 7, 8, and 9) produced a significantly larger number of words compared to participants with 5.8 or fewer switches (*n* = 26, obtained from combining nodes 3 and 4, χ^2^ = 0.29, *p* = 0.001). Furthermore, among participants with equal to or fewer than 5.8 switches, there is a further split (node 2), which also indicates that participants who had more switches in their data (above 3.2 switches, *n* = 17, represented in node 4) produced significantly more words than participants with fewer switches (at or below 3.2, *n* = 9, represented in node 3, χ^2^ = 11.451, *p* = 0.001). For participants who produced more than 5.8 switches, age of acquisition played a role, leading to the split point illustrated by node 5. Participants who produced words with a mean age of acquisition above 4.64 (*n* = 7, node 9) produced a larger total number of words compared to participants producing words of age of acquisition at or below the same value (*n* = 25, obtained from nodes 7 and 8, combined, χ^2^ = 11.816, *p* = 0.001). Furthermore, among the subgroup with age of acquisition values at or below 4.64, an additional split point is observed (node 6), further indicating that individuals who produced words with higher mean age of acquisition (above 4.14, *m* = 17, node 8) produced a larger total number of words compared to individuals who produced words of mean age of acquisition at or below 4.14 (*n* = 8, represented in node 7, χ^2^ = 10.505, *p* = 0.002).

Finally, Wilcoxon tests showed that the 35 individuals with AD that had scores below the norm in animal fluency produced significantly fewer switches and words of earlier age of acquisition than the remaining 23 individuals with AD that had within normal scores in animal fluency (see Table 2).

**TABLE 2:** Cross-validation of results with non-parametric tests.

	**AD below norm (*N* = 35)**	**AD within norm (*N* = 23)**	**Wilcoxon test**
Switches	*M* = 4.9; *SD* = 2.29	*M* = 10.12; *SD* = 3.19	*W* = 68.5, *p* = 0.0001
Age of acquisition	*M* = 4.23; *SD* = 0.6	*M* = 4.6; *SD* = 0.53	*W* = 257; *p* = 0.0211

*AD, Alzheimer’s disease; m, mean; sd, standard deviation; W, Wilcoxon test value.*

## Discussion

The purpose of this study was to understand factors that drive performance on animal fluency tasks in people with AD. Specifically, we wanted to identify whether and how variables, including mean cluster size, number of switches, and word properties, affect total number of words generated on this task. The main idea behind this study is that animal fluency is typically argued to involve language and executive functions. However, it is unclear whether which of the two functions is more relevant for people with AD, and what kind of linguistic (e.g., semantic level, phonological output lexicon, phonological assembly) and executive processes (e.g., mental set shifting, information updating and monitoring, and inhibition of responses) are most critical. Provided the current literature, we hypothesized that variables related to language processes may be more explanatory of total number of words in people with AD. However, we had some reservations regarding these hypotheses and stated that executive functions could also be involved, particularly for updating and monitoring responses.

Overall, our results indicate that both language and executive functions are involved in performing an animal fluency task. When we look at variables that can be extracted from fluency tasks, number of switches and age of acquisition appear as the most relevant factors. We reported these results with machine learning algorithms and we cross-validated them with a more commonly used test, namely, Wilcoxon tests. More importantly, the fact that both executive functions and language are involved was not unexpected (e.g., Shao et al., 2014). Nonetheless, the present study extends the previous work by showing how executive functions interact with linguistic variables. We observed that the number of switches (a variable that is argued to reflect executive functioning and particularly updating and monitoring of responses) discriminates people with AD who produce responses within the normal range for their age and education from those who fall below the normal range. For people whose responses fall within the normal range, age of acquisition (a linguistic variable argued to reflect processing at the level of the phonological output lexicon) was an important determinant of performance in the animal fluency task.

In animal fluency, number of switches can be thought of as marking efficient shifts between semantic subcategories. We found that people who produced more words also produced more switches. This pattern was also observed by Troyer et al. (1997) and others (e.g., Abwender et al., 2001). Troyer et al. (1997) argued that producing more switches indicated good executive functions and semantic knowledge. An alternative interpretation is that producing more switches results from difficulties with clustering, which could reflect impairments in semantic knowledge. That is, participants may produce more words and more switches because they cannot access concepts within the same subcategory. However, our data argue against this interpretation because cluster size was not selected as a relevant variable in the conditional inference trees.

In our data, participants with AD that generated more words produced both more switches and more words learned later in life. On this basis, we suggest that the number of switches indicates a successful strategy to retrieve more words. Hence, like Troyer et al. (1997), we suggest that producing more switches shows relatively good executive functioning (particularly updating and monitoring). In contrast to Troyer et al. (1997) though, we argue that producing more switches reflects retrieval from the phonological output lexicon rather than semantic processing *per se*. To give a more in-depth explanation: our data indicate that switching contributes more substantially to producing a large number of correct responses than clustering. Switching has been argued to index executive functions, and particularly to process regulating lexical retrieval, such as updating the criterion used to search words and keeping track of previous responses (e.g., Miyake et al., 2000; Mayr, 2002; Henry et al., 2004; Shao et al., 2014; Takács et al., 2014). Consequently, people with AD who have a greater cognitive capacity are likely to make more switches, and this behavior leads them to generate more words. However, people with AD who adopt the strategy of generating large clusters (i.e., retrieving as many items as possible within the same subcategory) are likely to rely more on lexical–semantic processing. This latter strategy is counterproductive, as individuals with AD tend to have difficulties in semantic processing (Monsch et al., 1992; Henry and Crawford, 2004) and also on the phonological output lexicon, as shown by our results on age of acquisition.

It may seem counterintuitive that age of acquisition explains the total number of words retrieved during an animal fluency task. This is because age of acquisition is more closely related to lexical processing than semantic processing (e.g., Whitworth et al., 2014; Alyahya et al., 2020), and animal fluency requires retrieval of words within the same semantic field. In fact, animal fluency is sometimes called “semantic fluency” for this reason (e.g., Henry and Crawford, 2004; Laws et al., 2010). Nonetheless, people with AD are known to have difficulties retrieving words that were learned later in life (e.g., Fisher et al., 2004; Choi, 2008; Cuetos et al., 2010). Therefore, it is reasonable to suspect that, in our sample, people with AD who produced more words (and were therefore less impaired) also produced more words learned later in life.

What in any case is difficult to explain is why other word properties that we argued relate to lexical–semantic processing did not emerge as important in our analyses. Looking at theories of age of acquisition in people with AD can shed light on this issue. There are multiple explanations of why age of acquisition is affected in people with AD (e.g., Cuetos et al., 2010). One account is that concepts learned earlier in life are more connected with other concepts, making them easier to access than concepts learned later in life (Brysbaert et al., 2000; Steyvers and Tenenbaum, 2005). This account implies that words learned earlier in life have stronger semantic connections than words learned later in life. Other scholars indicate that words learned earlier in life occupy greater space in the language network, making the language network less prone to learn new words or new word associations (Brown and Watson, 1987; Ellis and Lambon Ralph, 2000). This argument does not necessarily depend on semantic connections. For example, Brown and Watson (1987) suggest that words learned earlier, but not later, in life are stored in a complete form in the phonological output lexicon. The reason is that the number of words increases with age, requiring implementation of more efficient strategies for storage of lexical representations.

Importantly, proponents of the latter account indicate that frequency of exposure is not always relevant to explain performance on language tasks. Thus, age of acquisition rather than word frequency may be more relevant to determine performance in animal fluency (cf. Gilhooly and Watson, 1981). Likewise, other variables that typically correlate with frequency should not play a role in animal fluency either. Such variables include imageability, concreteness, and familiarity, which were not identified as relevant in our analyses or in other studies that used naming tasks (Nickels and Howard, 1994, 1995). Similar arguments could be used for the other variables we entertained in this study. That is, clusters, semantic association, and orthographic/phonological neighborhood did not emerge as relevant because these are variables that relate to the semantic system. Instead, our results seem to indicate that words learned earlier in life do not necessarily have more connections with other words (i.e., a stronger semantic representation), but a different (more unitary) representation than later acquired words, within the phonological output lexicon.

Regarding the limitations of our study, we only included one variable that has been argued to relate to executive functioning (i.e., switches), while the other variables we considered are more prone to reflect language processing. Unfortunately, it is hard to find other variables in an animal fluency task that would tap into different aspects of executive functioning and, even if we would do so, it would be hard to disentangle whether those variables are unique to one aspect of executive functioning, given the multifaceted character of executive functions (e.g., Miyake et al., 2000). Still, given a less strict perspective on the involvement of executive functions in fluency tasks, effects for phonological and orthographic neighborhood and length could relate to executive functioning and particularly inhibition (Henry et al., 2004; Takács et al., 2014). This is because words that are short tend to have more lexical neighbors (and therefore, to retrieve them many more words may have to be inhibited) than words that are long (e.g., soul–bowl, coal, dole vs. mountain–fountain). Nonetheless, and even though these word properties would not require conscious/controlled suppression of responses (cf. Miyake et al., 2000), none of these variables showed as relevant in the final results. Possibly, and following from the discussion above, neither neighborhood density measure appeared as relevant because performance in animal fluency tasks is driven by the functioning of the phonological output lexicon. Hence, we suggest that age of acquisition taps into the phonological output lexicon (not necessarily into semantics) and that words that are learned earlier in life have further weight in the language network regardless of word frequency or semantic connectedness (e.g., Brown and Watson, 1987; Ellis and Lambon Ralph, 2000). Thus, even though our participants produced more words learned earlier in life, the words they produced did not necessarily have more connections to other lexical representations, meaning that they did not require controlled suppression of responses or other aspects of executive function.

Future work could examine both separate measures of linguistic variables and executive functions and variables extracted from fluency tasks themselves (e.g., Shao et al., 2014). Although no single test can isolate specific aspects of executive functions, some additional tests that could be administered are the Wisconsin Card Sorting Test (Kimberg et al., 1997) to assess mental set shifting, the operation span (e.g., Turner and Engle, 1989) for information updating and monitoring, and Tower of Hanoi (e.g., Humes et al., 1997) for inhibition of responses. Another study could use other category fluency tasks to explore factors that are important in early lexical acquisition (e.g., Nickels and Howard, 1995). This type of work could provide further arguments to the relevance of the phonological output lexicon, as we argued here. For example, a new study could compare fluency of whole discrete objects and parts of objects (transportation means vs. parts of a bicycle, car), or compare fluency for typical items and less good exemplars or subcategories (e.g., animals vs. types of mammals, birds). If age of acquisition really drives performance, then it should appear as relevant, even in fluency tasks that are less prone to elicit these items, such as fluency of parts of objects or fluency of subcategories.

Another future avenue for research is to investigate similar questions in letter fluency tasks. This could be an interesting exercise, as letter fluency has been argued to rely less on semantic processing than animal fluency (Monsch et al., 1992; Henry and Crawford, 2004). Indeed, because participants need to retrieve words starting with the same letter, it can be argued that letter fluency taps further into lexical retrieval. Therefore, it is expected that word properties that relate to the output lexicon, such as frequency and phonological/orthographic similarity, to be more relevant in prediction of total word counts. Finally, determinants of performance on semantic and letter fluency tasks could be investigated in other neurological populations, such as people with a stroke, brain tumor, and other types of dementia. Such studies could assess whether the patterns reported here are unique to AD, and also to better understand the underlying disorders in other neurological populations.

## Conclusion

Two variables that relate to language and executive functioning (namely, age of acquisition and switches) were identified as predicting performance of people with AD in animal fluency tasks. People with AD and below-norm performance in animal fluency produced fewer switches and words with lower age of acquisition compared to people with AD and within-norm performance. These results are consistent with previous studies using different methodologies and with work stressing the role of age of acquisition in the language performance of people with AD. Above all, they indicate the relevance of the phonological output lexicon and information updating and monitoring to production of words in animal fluency.

## Data Availability Statement

All datasets generated for this study are included in the article/Supplementary Material.

## Ethics Statement

Ethical review and approval was not required for the study on human participants in accordance with the local legislation and institutional requirements. Written informed consent for participation was not required for this study in accordance with the national legislation and the institutional requirements. This study was done following the TalkBank Code of Ethics (https://talkbank.org/share/ethics.html).

## Author Contributions

All authors have contributed to the work, agree with the presented findings, and the work has not been published before nor is being considered for publication in another journal.

## Conflict of Interest

The authors declare that the research was conducted in the absence of any commercial or financial relationships that could be construed as a potential conflict of interest.
